# Probabilistic Short-Term Sky Image Forecasting Using VQ-VAE and Transformer Models on Sky Camera Data

**DOI:** 10.3390/jimaging12040165

**Published:** 2026-04-10

**Authors:** Chingiz Seyidbayli, Soheil Nezakat, Andreas Reinhardt

**Affiliations:** Department of Informatics, Clausthal University of Technology, 38678 Clausthal-Zellerfeld, Germany; soheil.nezakat@tu-clausthal.de (S.N.); reinhardt@ieee.org (A.R.)

**Keywords:** cloud motion forecasting, ground-based sky imaging, vector-quantized variational autoencoders, autoregressive transformer, uncertainty-aware prediction

## Abstract

Cloud cover significantly reduces the electrical power output of photovoltaic systems, making accurate short-term cloud movement predictions essential for reliable solar energy production planning. This article presents a deep learning framework that directly estimates cloud movement from ground-based all-sky camera images, rather than predicting future production from past power data. The system is based on a three-step process: First, a lightweight Convolutional Neural Network segments cloud regions and produces probabilistic masks that represent the spatial distribution of clouds in a compact and computationally efficient manner. This allows subsequent models to focus on the geometry of clouds rather than irrelevant visual features such as illumination changes. Second, a Vector Quantized Variational Autoencoder compresses these masks into discrete latent token sequences, reducing dimensionality while preserving fundamental cloud structure patterns. Third, a GPT-style autoregressive transformer learns temporal dependencies in this token space and predicts future sequences based on past observations, enabling iterative multi-step predictions, where each prediction serves as the input for subsequent time steps. Our evaluations show an average intersection-over-union ratio of 0.92 and a pixel accuracy of 0.96 for single-step (5 s ahead) predictions, while performance smoothly decreases to an intersection-over-union ratio of 0.65 and an accuracy of 0.80 in 10 min autoregressive propagation. The framework also provides prediction uncertainty estimates through token-level entropy measurement, which shows positive correlation with prediction error and serves as a confidence indicator for downstream decision-making in solar energy forecasting applications.

## 1. Introduction

The use of solar panels, also known as photovoltaic (PV) systems, is growing very fast [1]. Because of this, accurate short-term forecasts of the solar radiation received on the ground are more important than ever [2]. The dynamic and frequently changing nature of clouds introduces significant variability in solar irradiance, making solar power generation less predictable under cloudy conditions. Rapid changes in cloud cover can cause abrupt fluctuations in solar irradiance; therefore, accurate forecasts of cloud motion are essential to understand PV generation better and use this knowledge to balance electricity supply and demand [3,4].

Sky cameras are useful tools to capture timestamped images of cloud fields. These observations are widely used in both research and operational settings for short-term cloud-motion tracking and solar irradiance forecasting [5,6]. From a remote sensing perspective, all-sky cameras constitute a form of ground-based passive optical remote sensing, in which data quality is affected by instrument-specific characteristics such as wide-angle geometric distortion inherent to fisheye optics, radiometric variations induced by atmospheric aerosol scattering, and changes in solar elevation angle throughout the day [7,8]. While satellite-based cloud products offer broad spatial coverage but are limited by coarse temporal resolution, ground-based sky cameras provide higher-temporal-resolution imagery over a localized domain (typically on the order of a few kilometers), serving as a complementary observational modality with sub-minute temporal sampling directly relevant to site-specific PV energy management and intra-hour nowcasting [9,10]. However, even with high-resolution sky-camera observations, predicting the subsequent evolution of cloud fields remains challenging. This difficulty stems from the fact that cloud evolution is governed by nonlinear, multiscale atmospheric dynamics in which small differences in initial conditions can lead to substantially different outcomes, thereby limiting forecast predictability [11].

In previous studies using sky cameras, cloud motions have often been estimated using optical flow in conjunction with physics-based advection models [5]. Optical flow is an image processing approach that calculates the motion vector field between successive frames under the assumption of approximate brightness constancy. However, these traditional methods can break under rapidly changing conditions (e.g., cloud formation, deformation, or dissipation), as they ignore non-stationary deformations and dynamic changes in cloud geometry, leading to unstable or inconsistent motion estimates [12]. Another limitation of these conventional methods is that the degree of certainty associated with their predictions is often not reported, which restricts their use in applications that call for risk-aware decision-making. This limitation motivates the incorporation of uncertainty quantification so that forecasts can be accompanied by an explicit and interpretable confidence indicator.

The development of deep neural networks has significantly improved spatiotemporal forecasting capabilities by enabling models to learn complex spatial and temporal dependencies across high-dimensional environmental data [13]. Neural network-based convolutional recurrent models, including the Convolutional Long Short-Term Memory (ConvLSTM) architecture, have been widely used for cloud movement and weather forecasting [14]. However, ConvLSTM-based methods often produce smoothed or blurry predictions at longer forecast lead times, indicating limitations in preserving fine spatial structure and detailed motion dynamics [15]. Another limitation is that deterministic pixel-level losses often yield blurry predictions, as the model is penalized equally for all deviations from the ground truth and thus learns to produce conservative, mean-like outputs rather than sharp spatial structure [16]. Also, small discrepancies at the beginning of the forecast have been reported to grow into large errors later on [17]. Most of these pixel-to-pixel models also struggle to explain the uncertainty of their own predictions [16,18,19].

Rather than predicting how pixel patterns move over time directly (i.e., in pixel space), learned latent representations can be used to model the future evolution of cloud movements. A suitable way to encode images into compact discrete latent representations is through the use of Vector-Quantized Variational Autoencoders (VQ-VAEs) [20]. The resulting discrete representation reduces spatial complexity and enables sequence-based temporal modeling [21]. Transformer-based autoregressive models have shown robust performance in video prediction tasks due to their ability to model long-term dependencies [22,23]. Uncertainty quantification is critical in renewable energy forecasting because it enhances the reliability of forecasts by presenting probability intervals for possible outcomes and supports risk-aware operational decisions [24]. Accordingly, uncertainty-aware forecasts enable better decision-making by providing richer information than single-point forecasts [25].

Existing cloud-motion and irradiance nowcasting pipelines often rely on assumptions (e.g., appearance constancy or slowly varying cloud structure) that may not hold true under rapid cloud formation, deformation, or dissipation, leading to degraded predictive performance. To mitigate these limitations, this work adopts a modular framework that separates (i) cloud-field characterization, (ii) a compact state representation, and (iii) temporal evolution modeling, with the aim of improving robustness under dynamic cloud conditions. The specific architectural design choices—namely CNN-based segmentation for cloud mask extraction, latent tokenization via VQ-VAE, and Transformer-based temporal prediction—are described in detail in Section 3. Notably, predicting binary cloud masks rather than full RGB images allows the model to focus on physically relevant cloud dynamics while reducing representational complexity [26,27].

## 2. Related Work

Short-term cloud motion forecasting has been studied from multiple perspectives, including image-based sensing, cloud segmentation, and spatiotemporal prediction models. In the following sections, we review the most significant works in sky-camera analysis, deep learning approaches to cloud-pattern segmentation, and representative state-of-the-art methods for predicting cloud movement over time.

### 2.1. Sky Camera Cloud Observation

For monitoring clouds at a local scale, sky cameras offer high-resolution observations that are more accessible and cost-effective than satellite-based imaging. Their ability to capture rapid changes at a low cost makes them ideal for short-term tasks. Early studies demonstrated the feasibility of estimating cloud motion vectors from sky images using optical flow and geometric projection models [5,28]. More recent works have focused on using sky imagery to improve solar irradiance forecasting [29,30]. Research in this area suggests that accurately estimating cloud motion from sky images is a powerful tool for reducing the uncertainty of short-term solar power [22]. Also, studies based on all-sky camera observations have shown that high-resolution cloud segmentation and cloudiness retrieval at the local scale can complement satellite products and improve the understanding of short-term cloud variability [31]. Advances in data-driven methods, particularly deep learning-based cloud segmentation from all-sky images, further highlight the potential of sky cameras for robust cloud monitoring under diverse atmospheric conditions [32,33]. However, many traditional methods still rely on handcrafted features such as color thresholds, edge patterns, or optical-flow vectors, or physical assumptions that often struggle when faced with complex cloud behavior including cloud growth or dissipation, multi-layer motion, or rapid appearance changes caused by light, all of which occur frequently in real-world sky camera observations [34,35].

### 2.2. Cloud Segmentation Using Deep Learning

Correctly segmenting images into cloud and non-cloud areas is crucial, as even small errors in identifying cloud patterns can lead to significant inaccuracies in the final prediction [27,33]. Datasets such as Singapore Whole sky Nychthemeron Image Segmentation (SWINySEG) [36] provide the pixel-level cloud–sky annotations necessary for supervised training, laying the foundation for a fair comparison of cloud segmentation models. Classical threshold-based and color-space methods often fail in challenging illumination conditions such as sunrise, sunset, or thin cloud layers. To address these limitations, Convolutional Neural Networks (CNNs) have been increasingly adopted for cloud–sky segmentation tasks [36]. Lightweight CNN architectures have gained traction because they are well-suited for the limited processing power of edge devices and embedded systems. Models such as U-Net variants and compact encoder–decoder networks have shown strong performance in binary cloud masking while maintaining low computational cost [36]. In this context, Lightweight CNN inspired by UCloudNet is attractive, as it is developed to maintain cost efficiency while ensuring high accuracy and has shown that this architecture achieves both high segmentation accuracy and computational efficiency for cloud mask extraction from sky images [37].

### 2.3. Spatiotemporal Forecasting of Cloud Dynamics

Because cloud fields exhibit variability in both morphology and motion, accurate forecasting of their evolution requires models capable of capturing complex spatiotemporal dependencies. Early methods applied optical flow and motion extrapolation directly to cloud masks or image intensities [38]. While these methods are computationally efficient, they are limited in predicting nonlinear cloud motion. Recent developments in deep learning have led to major improvements in spatiotemporal forecasting, offering a level of precision that was previously difficult to achieve. Recurrent neural network (RNN)-based architectures, particularly ConvLSTM models [14], have been extensively applied to precipitation nowcasting and cloud motion prediction [39,40,41]. These models effectively capture temporal patterns, but when trained with deterministic pixel-based loss functions, they tend to produce fuzzy predictions, especially in multimodal scenarios where multiple possible future scenarios exist. For cloud boundary prediction, this fuzziness can compromise the spatial accuracy of the predicted cloud masks and contribute to error accumulation over longer prediction horizons. Transformers have emerged as a powerful alternative for sequence modeling, owing to their ability to capture long-range dependencies through self-attention mechanisms. Their effectiveness has been demonstrated across diverse domains, including video modeling, time-series forecasting, and audio processing [42,43]. Several studies have applied transformer-based architectures to video prediction and weather forecasting tasks, showing improved temporal consistency compared to recurrent models [19,44]. Despite their strengths, applying transformers directly to high-resolution image prediction remains computationally expensive.

### 2.4. Latent-Space and Token-Based Forecasting

To reduce computational cost, recent work has shifted toward latent-space forecasting as a more efficient approach. Several methods exist for representing images as discrete tokens including patch-based tokenization and learned codebooks, but VQ-VAE has gained prominence because its learned discrete latent codes offer a compact representation that is well suited for high-resolution image modeling and autoregressive sequence prediction [20,45]. Accordingly, sequence models can operate directly on these tokens rather than in high-dimensional pixel space. This strategy has been successfully applied to video generation and prediction using autoregressive transformers [46]. Latent token forecasting excels at cloud prediction because it tracks essential structural patterns while ignoring irrelevant pixel-level noise. Recent studies suggest that working with tokens helps maintain a clear spatial structure while still allowing the model to explore different possible outcomes through probabilistic sampling [46,47,48].

### 2.5. Physics-Informed Temporal Modeling

A common issue with forecasting is that models can start to ignore basic physical constraints during longer, multi-step forecasts. To overcome this issue, physics-informed neural networks embed physical laws into the model’s training objective by enforcing the governing differential equations, which helps constrain solutions and improve robustness, especially when data are limited [49]. The Physics-informed Cell (PhyCell) framework introduces a recurrent cell structure inspired by physical evolution equations and a prediction correction paradigm [50] (drawing on data-assimilation ideas), enabling partial differential equation (PDE) constrained prediction in a learned latent space and improving long-term forecasting behavior and robustness to missing inputs [50].

Hybrid models that combine deep learning with physics-guided components have also shown practical benefits in Earth-system prediction, for example by integrating a process-based ecosystem model with a long short-term memory to improve accuracy while retaining physical interpretability [51]. We see that existing methods often focus on either pixel-level prediction or latent modeling, but they frequently lack a framework for explicit physical guidance; moreover, physical laws may not apply directly in pixel space for generic video/cloud imagery, motivating latent spaces where physical and residual factors can be disentangled [50]. There are limited approaches that simultaneously handle computational efficiency, physical laws, and uncertainty in the context of short-term cloud motion prediction; in this direction, operator-learning surrogates highlight how offline training can enable fast online inference (a simple forward pass) once trained [52]. The proposed framework addresses these gaps by merging efficient cloud segmentation with transformer-based forecasting and physical laws to predict cloud movement.

## 3. Methodology

**Figure 1:**
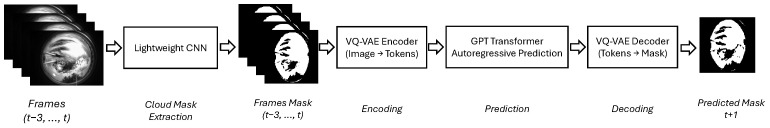
The general architecture of the pipeline consisting of a lightweight CNN, VQ-VAE, and an autoregressive Transformer.

The proposed cloud motion nowcasting system given in Figure 1 operates as a three-stage sequential pipeline: (1) cloud segmentation via a lightweight CNN to produce probability masks, (2) compression into discrete token sequences using a VQ-VAE, and (3) autoregressive temporal forecasting with a Generative Pre-trained Transformer (GPT)-style transformer. Each stage is trained independently, with outputs from earlier modules serving as inputs to subsequent ones.

By predicting cloud probability masks rather than raw RGB images, the system focuses on meteorologically relevant cloud geometries while filtering out illumination variations and background noise [53,54]. The VQ-VAE further abstracts these masks into a discrete token vocabulary, enabling efficient transformer-based sequence modeling and preventing physically implausible predictions during multi-step rollout [20,45]. The following subsections detail each component’s architecture and training procedure.

### 3.1. Cloud Detection Using a Lightweight CNN

Cloud detection is performed using a lightweight CNN inspired by the UCloudNet architecture [37] but substantially simplified for computational efficiency. The primary objective of this network is to transform raw sky images into continuous pixel-wise cloud probability masks. Unlike binary (0/1) segmentation, these probability masks produce a value in the range [0,1] for each pixel, where 0 represents clear sky with certainty, 1 represents cloud with certainty, and intermediate values represent ambiguous or partial cloud coverage, such as thin cirrus layers, cloud edges, or semi-transparent regions. This continuous representation provides smoother and more realistic spatial transitions compared to hard binary segmentation, particularly near cloud boundaries and in regions with thin cloud structures [36]. While the original UCloudNet employs a deep U-Net architecture with multiple encoder–decoder levels, the proposed network is simplified to a single-level minimal encoder–decoder structure to reduce computational cost. The model consists of only four convolutional layers and contains approximately ~14,300 parameters—roughly 23 times fewer than UCloudNet’s ~330,000 parameters. This reduction in model complexity makes real-time inference possible even on resource-constrained devices.

**Figure 2:**
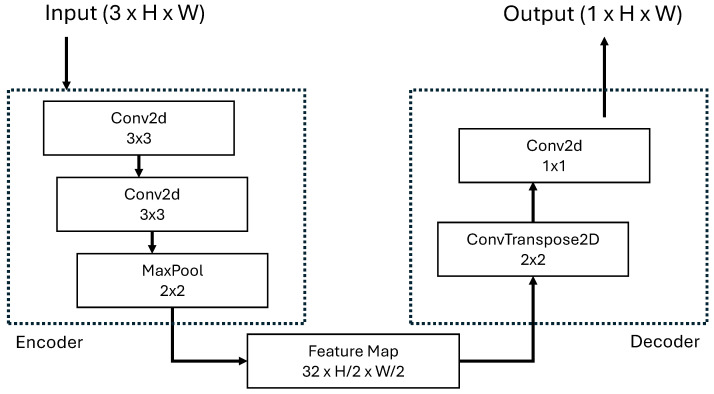
LightweightCNN architecture, inspired by the UCloudNet architecture that converts the input image into a binary mask image of the same size.

As shown in Figure 2 the encoder comprises two successive 3×3 convolutional layers (with padding = 1 to preserve spatial dimensions) followed by a single 2×2 max-pooling operation that downsamples the spatial resolution by a factor of two. Each convolutional layer uses 32 feature channels and ReLU activation. The decoder restores the original spatial resolution through a learned transpose convolution layer (2×2 kernel, stride = 2) followed by a 1×1 convolutional layer that projects the 32 feature channels into a single output channel. Transpose convolution is preferred over bilinear interpolation, as it provides learnable upsampling weights, enabling the model to generate sharper cloud boundaries. The model accepts input images of size H×W×3 in RGB format where H×W stays for image dimension of the resized at 256×256; however, since grayscale images are used in practice, all three channels contain identical values. The 3-channel structure has been retained because the standard PyTorch vision models expect pre-trained weights with 3-channel RGB inputs. The output consists of logit values of size H×W×1, which are converted to probability masks in the range [0,1] via sigmoid activation. The network is trained using Binary Cross-Entropy with Logits Loss (BCEWithLogitsLoss) loss to optimize pixel-wise classification accuracy. Several architectural components present in UCloudNet have been deliberately removed based on task-specific considerations. First, skip connections—a hallmark of U-Net architectures—are omitted because cloud–sky segmentation is a relatively simple two-class problem where cloud regions exhibit high internal homogeneity and are easily distinguishable from the sky background, unlike complex multi-class medical imaging tasks that typically motivate U-Net designs. While this simplification introduces minor blurring (1–2 pixels) at cloud edges, this loss is negligible in the overall pipeline since the subsequent VQ-VAE encoder applies 8× spatial downsampling. Second, residual connections are excluded because the shallow depth of the network (only four convolutional layers) does not pose a risk of vanishing gradients, rendering residual paths unnecessary. Third, batch normalization layers are removed to reduce inference overhead and avoid batch-size dependency, which is problematic in real-time single-image processing scenarios; simple input normalization (scaling images to [0,1]) provides sufficient training stability for this lightweight architecture. This minimalist design philosophy prioritizes deployment efficiency without compromising the core segmentation capability required for cloud motion nowcasting. The resulting model achieves a favorable trade-off between computational cost and task-specific performance, making it suitable for edge deployment and real-time applications.

### 3.2. Discrete Latent Representation with VQ-VAE

**Figure 3:**
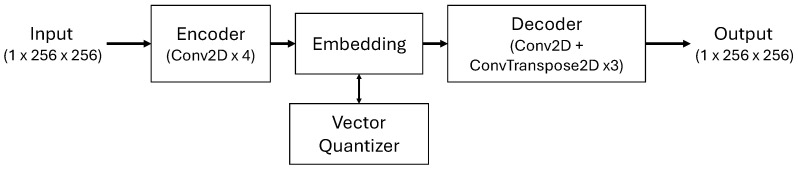
VQ-VAE model architecture.

To represent cloud masks in a compressed and structured format, a customized VQ-VAE architecture is used [20]. The primary function of this module is to convert high-resolution spatial cloud masks (H×W pixel array) into discrete symbolic token sequences; thus, the subsequent transformer-based prediction model can operate on meaningful cloud structure patterns selected from a limited codebook rather than continuous pixel values. The VQ-VAE model architecture is given in Figure 3. The encoder architecture reduces the binary cloud mask by a factor of 8 through convolutional layers and produces a compact latent representation for each mask. The architecture produces a 64-dimensional continuous feature vector for each spatial location using three consecutive strided-convolution layers.

The 8× spatial downsampling filters out local pixel noise in cloud masks, preserving only large-scale cloud structures (cloud clusters, boundaries, and holes); this allows the transformer model to operate on fewer tokens and reduces computational cost by approximately 64 times. The encoder output is converted into discrete symbols using a fixed-bit embedding table, which is a learnable codebook consisting of 1024 entries, each being a 64-dimensional vector. The reason for selecting this codebook size is that usage examples are also available in the literature [45]. Furthermore, the ablation study results for codebooks of varying sizes and the rationale for the selected configuration are provided in Section 5. For each spatial location, the encoder output is mapped to the nearest vector according to the Euclidean distance in the codebook, and as a result of this process, a token index is obtained for each spatial location. Consequently, each cloud mask is represented as a token map of size H/8×W/8 and corresponds to a selected cloud structure prototype from the codebook. The obtained quantified latent representation values are converted back to the original cloud mask using a decoder with three consecutive transpose convolution (deconvolution) layers. This restructuring is necessary because the transformer operates in a compressed discrete symbol space for computational efficiency but visualization requires interpretable pixel-level cloud masks for comparative evaluation with real data.

### 3.3. Temporal Forecasting Using an Autoregressive Transformer

To model cloud motion, a GPT autoregressive transformer architecture that learns the temporal evolution of discrete token sequences is used [21]. The main task of this module is to predict cloud masks at the future time step at the token level, taking token sequences at the past time step as input (e.g., predicting the next 8 masks from the last 4 cloud masks). At each time step, the cloud mask is converted to H/8×W/8 tokens by the VQ-VAE encoder. This 2D token map is flattened into a 1D sequence. If 4 past time steps are used, the total input length is 4 × (H/8×W/8) tokens. Each token is an index value selected from the VQ-VAE codebook. Our model uses a decoder-only transformer architecture; this is the same approach used in GPT and similar autoregressive language models. This design includes only a causal self-attention mechanism; that is, each token can only access at the tokens preceding it and has no access to future tokens.

Each token index is first converted into a 256-dimensional continuous vector via a learnable embedding lookup table—a 1024×256 weight matrix where the token index serves as a key to retrieve the corresponding row vector. Since transformers do not naturally carry sequence information, a learnable positional encoding is added to each token. Learnable encoding is preferred over fixed sinusoidal encoding because the spatiotemporal structure of cloud sequences differs from that of natural language, and it is more appropriate for the model to learn this structure itself.

Concretely, a learnable positional embedding matrix of shape $\left(T+K\right)\cdot S\times d_{\mathrm{model}}$ is employed, where $S=\frac{H}{8}\times \frac{W}{8}$ is the number of tokens per frame and $d_{\mathrm{model}}=256$ is the embedding dimension. Each of the (T+K)·S positions receives a unique trainable vector that jointly encodes both the spatial location of the token within its frame and its temporal offset across frames. Although the 2D token map is serialised into a 1D sequence via raster-scan (row-major) order, the learned embeddings implicitly recover spatial proximity. To verify this, pairwise cosine similarities between all token embeddings within a single frame are computed on the trained model: tokens that are direct spatial neighbours in the $\frac{H}{8}\times \frac{W}{8}$ grid (4-connected, Manhattan distance =1) yield a mean cosine similarity of 0.051, whereas tokens separated by Manhattan distance ≥5 yield only 0.004, giving a locality ratio of 12.4×. This demonstrates that spatial context is preserved during autoregressive sequence modelling despite the 1D serialisation.

The model consists of 6 identical transformer layers, and each layer contains multi-head causal self-attention, feed-forward network and residual connections and, layer normalization. The ablation study regarding the selection of the number of layers is presented in Section 5. In encoder–decoder transformers, encoder layers use bidirectional full attention, while decoder layers use causal attention. Since the proposed model has a decoder-only architecture, causal masking is applied in all layers. It provides critical advantages in causal masking, temporal consistency, long-range dependencies [19], and working at the token level instead of the pixel level [47,48].

Operating at token level is also crucial for performance: If the model operated at the pixel level, the attention matrix would be ${\left(H\times W\right)}^{2}$ in size. However, since the model operates at the token level, both the height and width dimensions are reduced by a factor of 8. In this case, the total matrix size of

(1) $${\left(\frac{H}{8}\times \frac{W}{8}\right)}^{2}=\frac{{\left(H\times W\right)}^{2}}{64^{2}}$$

is obtained. Therefore, the attention matrix is $64^{2}=4096$ times smaller compared to the pixel level. This also shows that the calculation cost and duration have been reduced by a factor of 4096.

### 3.4. Autoregressive Multi-Step Forecasting Strategy

Our framework employs an autoregressive forecasting approach where predictions are generated iteratively in a sliding-window manner. Given an initial temporal context of four consecutive cloud mask token sequences (frames at t−3,t−2,t−1,t), the transformer predicts the token sequence for the next time step (t+1). This predicted sequence is then incorporated into the context by appending it and dropping the oldest frame, creating a new context window (frames at t−2,t−1,t,t+1) for predicting t+2. This recursive process continues for the desired forecast horizon, enabling multi-step predictions without requiring ground-truth future observations during inference.

## 4. Data Selection and Model Training

The complete training pipeline consists of three sequential stages, each optimizing a distinct component of the forecasting system. The lightweight CNN model was trained using the SWINySEG dataset [36]. Then, binary mask images of the Clausthal All Sky Camera Recordings (CASCAR) dataset [55], which has 5 s sampling rate, were extracted using this pre-trained model, and other models were trained using this dataset.

### 4.1. Stage 1: Cloud Segmentation Network

The lightweight CNN is trained end-to-end to produce pixel-wise cloud probability masks from raw grayscale sky images. Training data consists of 6768 image-mask pairs from the SWINySEG dataset, where masks are binary ground-truth labels (0 = clear sky, 1 = cloud) created through the annotation procedure described in Section 3.

Images are randomly shuffled at each epoch to prevent overfitting to temporal ordering. No explicit data augmentation (e.g., rotation and flipping) is applied, as fisheye sky images have inherent rotation invariance that the model must learn.

The model is trained using BCEWithLogitsLoss, which combines a sigmoid activation with the binary cross-entropy criterion in a numerically stable form:

(2) $$L_{\mathrm{seg}}=-\frac{1}{HW}\sum_{i}\left[y_{i}\log{\hat{p}}_{i}+\left(1-y_{i}\right)\log\left(1-{\hat{p}}_{i}\right)\right],\;$$

where $y_{i}\in \left\{0,1\right\}$ is the ground-truth cloud label and ${\hat{p}}_{i}$ is the predicted cloud probability at pixel *i*. This loss is well-suited for binary cloud/sky segmentation because it directly models per-pixel binary probabilities and operates on logits for improved numerical stability, leading to reliable and stable optimization in dense prediction tasks. The Adam optimizer was used for model training, and a learning rate of $10^{-3}$ was employed.

### 4.2. Stage 2: VQ-VAE for Discrete Latent Representation

The VQ-VAE is trained to compress cloud probability masks (output from Stage 1) into discrete token sequences while preserving structural cloud patterns. Training data consists of temporal sequences with a sliding window approach: each sample contains $T_{\mathrm{context}}+T_{\mathrm{pred}}$ consecutive frames (e.g., 4 context + 1 prediction = 5 frames total), where frames are sampled with stride 5 s to capture temporal dynamics at sufficient time scales. All frames within each temporal sequence (both context and prediction frames) are processed independently through the VQ-VAE to maximize data utilization.

Training continues with the standard VQ-VAE objective combining L1 reconstruction loss and commitment loss [20], where the code book is updated using an exponential moving average (EMA) instead of direct backpropagation for numerical stability. The model converges when the reconstruction quality stabilizes and the codebook usage reaches sufficient diversity (measured by complexity). The final model is frozen and reused as a fixed encoder/decoder pair for the next stage.

The codebook size of K=1024 and the commitment cost β=0.25 follow the original VQ-VAE formulation of [20], in which β was identified as the empirically optimal value that prevents codebook collapse while maintaining a useful gradient signal for the encoder. The hidden channel width of 128 and latent dimension of 64 were chosen to balance representational capacity against computational cost for single-channel 256×256 inputs.

### 4.3. Stage 3: Autoregressive Transformer for Temporal Forecasting

The GPT-based transformer is trained to predict future token sequences given past context, conditioned on the frozen VQ-VAE encoder. The VQ-VAE weights from the previous Stage 2 are loaded and frozen to prevent catastrophic forgetting of the learned discrete representation. Training data consists of temporal sequences where context frames (T=4) and target frames (K=1 or more) are encoded into discrete tokens via the frozen VQ-VAE encoder.

For each training sample, context and target frames are first encoded to discrete tokens via the frozen VQ-VAE encoder. These token sequences are concatenated into a single flat sequence of length *L*, then split into input $x=[z_{1},\ldots ,z_{L-1}]$ and target $y=[z_{2},\ldots ,z_{L}]$ for standard teacher forcing: the model predicts the next token at each position given all previous tokens. This formulation trains the transformer to autoregressively generate token sequences that, when decoded by the VQ-VAE decoder, correspond to future cloud masks. The causal attention mask ensures that predictions at position *i* only depend on tokens $z_{1},\ldots ,z_{i}$, preventing information leakage from future time steps.

The GPT Transformer is trained with standard autoregressive cross-entropy loss over the discrete token vocabulary V:

(3) $$L_{\mathrm{GPT}}=-\frac{1}{L-1}\sum_{t=1}^{L-1}\log P_{\theta }\;\left(x_{t}|x_{<t}\right),\;$$

where $x_{t}\in V$ is the ground-truth token at position *t* and L=(T+K)·S is the total sequence length, with *T* context frames, *K* prediction frames, and *S* tokens per frame. The autoregressive cross-entropy objective is the standard training criterion for GPT-style sequence models [56], and transfers directly to discrete VQ token sequences. The architecture consists of 6 transformer layers, 512 hidden dimensions, and 8 attention heads (each operating in a 64-dimensional subspace), where the hidden dimensions and attention head configuration follow the design principles of [21], and the number of layers is determined based on the ablation study presented in Section 5.6. A dropout rate of 0.1 is applied throughout, consistent with the original transformer implementation of [21].

## 5. Evaluation of the System

The objective of this evaluation is to verify whether the proposed pipeline can produce short-term cloud-mask forecasts from ground-based sky-camera sequences with high accuracy, and whether its probabilistic sampling provides uncertainty estimates that are informative about prediction errors. We focus on three practical questions that can be answered directly by means of the experiments:

(Q1) Following the standard next-frame prediction protocol in the video prediction literature [57], can the model accurately predict the next cloud mask from four past frames on unseen sequences?
(Q2) Does the model remain usable under recursive rollout, i.e., does forecast quality degrade gradually rather than collapsing over multiple steps?
(Q3) Do higher uncertainty values tend to coincide with higher prediction error, indicating that the uncertainty estimates are meaningful for identifying less reliable prediction?

### 5.1. Sky Image Acquisition and Pre-Processing

Data for this study has been obtained from the CASCAR dataset [55]. The dataset was collected via a ground-based automated sky observation system which uses a roof-mounted sky-facing camera. The system consists of an Oculus All-Sky Camera 150° [58] camera and Oculus 180 Lens [59] (Starlight Xpress Ltd., The Old Dairy, Allanbay Park, Howe Lane, Binfield, Berkshire RG42 5QA, UK), controlled via the Instrument Neutral Distributed Interface (INDI) protocol. Image capture was performed during daylight hours, with sunrise and sunset times calculated daily based on geographic location information using astronomy libraries. The dataset has been sampled at a high temporal resolution, with between four and twelve images per minute.

This sampling strategy was adopted to capture the short-term evolution of cloud dynamics and, in particular, to preserve the temporal continuity of fast-moving cloud motions. To ensure consistent image quality under variable lighting conditions, an automatic exposure optimization algorithm was run every hour starting from sunrise that day. The algorithm performs an iterative search to converge the normalized pixel intensity of the image’s 99th percentile to a target value of 0.60. This approach adaptively responds to atmospheric lighting conditions that change from morning to noon to evening, depending on the sun’s altitude, preventing both overexposure (saturation) and underexposure. Each image has a size of 800×600 pixels (width × height) in the dataset.

### 5.2. Experimental Setup

**Figure 4:**
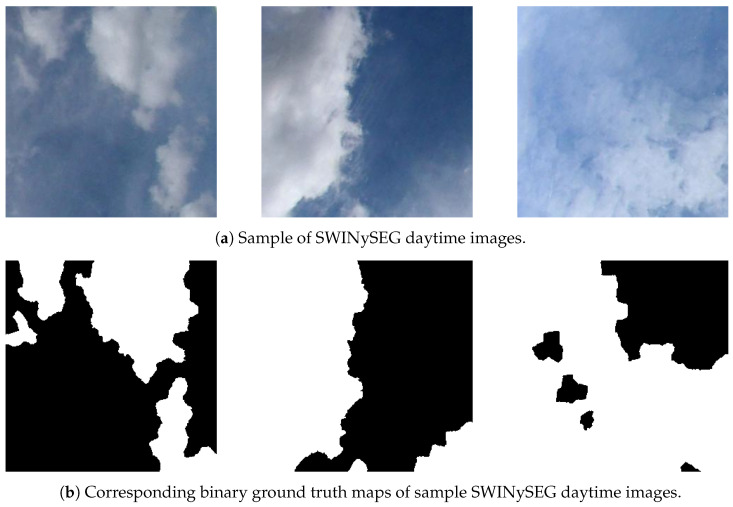
Original and binary mask view of data from SWINySEG dataset [36].

A total of 103,304 images were used in the study which were captured on different days and different hours of the day (152 different sequences) by considering various cloud densities and the speed of change of the clouds’ movement behavior [60]. When selecting these data, care was taken to choose partly cloudy images where clouds could be segmented and their movements predicted, rather than completely cloud-free or completely cloudy images from the dataset. Each sequence consists of binary cloud probability masks generated by the cloud segmentation model described in Section 3. The training data follows the annotation principles of publicly available ground-based sky datasets such as SWINySEG [36], ensuring compatibility with existing benchmarks [37]. As shown on Figure 4 in SWINySEG, each fisheye sky image is paired with a manually annotated binary ground truth mask where each pixel is labeled as cloud or clear sky.

The evaluation focuses on short-term cloud forecasting, which is crucial for managing PV power variability and supporting grid-balancing operations, where the model predicts future cloud masks based on a fixed number of past frames. In the study 15% (23 sequences) and 5% (7 sequences) of the data used were used as training and validation data, respectively. The remaining data (122 consecutive frames, equaling 82,931 images) were used to measure model performance, while the model was required to predict the next frame in the sequence.

All models were trained and evaluated on a server equipped with two 16-core AMD EPYC 7282 processors and Nvidia A100 80 GB GPU, running CUDA 12.4.

### 5.3. Evaluation Metrics

To evaluate model performance, the metrics Intersection over Union (IoU), F1-score, pixel accuracy, Mean Absolute Error (MAE), Mean Squared Error (MSE), and Structural Similarity Index Measure (SSIM) are employed, which are widely used in the remote sensing and image segmentation literature. Before calculating the binary classification metrics, both the predicted masks and the ground-truth masks are passed through a threshold of t = 128/255 to obtain a binary representation.

The IoU quantifies the spatial overlap between predicted and ground-truth cloud regions. Unlike accuracy-based measures, it is largely unaffected by class imbalance, making it particularly suitable for scenes where cloud coverage is sparse:

(4) $$IoU=\frac{\left|P\cap G\right|}{\left|P\cup G\right|},$$

where *P* and *G* denote the sets of predicted and ground-truth cloud pixels, respectively.

The F1-score, equivalent to the Dice coefficient in binary segmentation, accounts for both false positives and false negatives and provides a balanced measure of overlap between the predicted and reference masks:

(5) $$F1=\frac{2|P\cap G|}{\left|P|+|G\right|}.$$

Pixel accuracy measures the fraction of correctly classified pixels across the entire frame:

(6) $$Accuracy=\frac{N_{\mathrm{correct}}}{N_{\mathrm{total}}},$$

where $N_{correct}$ is the number of correctly classified pixels and $N_{total}$ is the total number of pixels in the image.

MAE and MSE capture pixel-level intensity differences between the predicted and ground-truth images. MAE computes the mean absolute deviation and is more robust to outliers, while MSE penalizes larger errors more heavily by squaring the residuals:

(7) $$MAE=\frac{1}{N}\sum_{i=1}^{N}\left|\hat{y}i-y_{i}\right|,$$

(8) $$MSE=\frac{1}{N}\sum {i=1}^{N}{\left({\hat{y}}_{i}-y_{i}\right)}^{2},$$

where ${\hat{y}}_{i}$ and $y_{i}$ are the predicted and ground-truth intensity values at pixel *i*, and *N* is the total number of pixels.

Finally, SSIM evaluates perceptual similarity by jointly considering luminance, contrast, and structural information between two images:

(9) $$SSIM\left(\hat{I},I\right)=\frac{\left(2\mu_{\hat{I}}\mu_{I}+c_{1}\right)\left(2\sigma_{\hat{I}I}+c_{2}\right)}{\left(\mu_{\hat{I}}^{2}+\mu_{I}^{2}+c_{1}\right)\left(\sigma_{\hat{I}}^{2}+\sigma_{I}^{2}+c_{2}\right)},$$

where $\hat{I}$ denotes the reconstructed image and I the reference image, $\mu_{\hat{I}}$ and $\mu_{I}$ are the mean intensities of $\hat{I}$ and I, respectively, $\sigma_{\hat{I}}^{2}$ and $\sigma_{I}^{2}$ are their corresponding variances, $\sigma_{\hat{I}I}$ the covariance between the two images, and $c_{1}$, $c_{2}$ are small stabilizing constants included to avoid division by zero. SSIM ranges from −1 to 1, with a value of 1 indicating perfect structural agreement.

### 5.4. Training Performance

**Figure 5:**
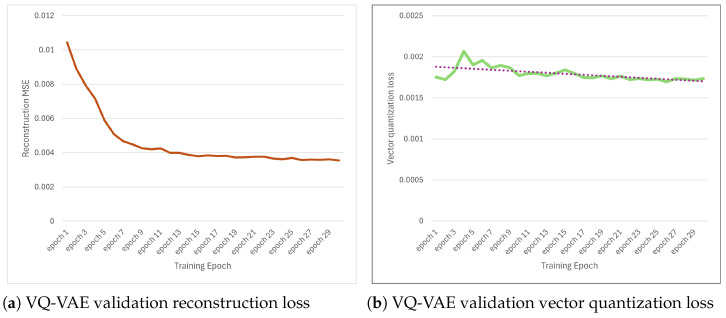
Training convergence of the VQ-VAE tokenizer. (**a**) Reconstruction loss demonstrates improved fidelity of cloud mask reconstruction over training epochs. (**b**) Vector quantization loss indicates stabilization of the learned discrete codebook.

Figure 5 illustrates the convergence behavior during the training of the VQ-VAE model, which constitutes the second stage of the pipeline. Two complementary loss components are observed: (a) the reconstruction loss $L_{\mathrm{recon}}$, which measures how accurately the decoder recovers the original cloud probability mask from the quantized tokens, and (b) the commitment loss $L_{\mathrm{VQ}}$, which ensures that the encoder outputs remain close to the code book embeddings.

Figure 5a shows that the MSE value of the reconstruction rapidly decreases from 0.0105 to approximately 0.0045 in the first 7 cycles, then gradually approached the final value of 0.0037 in the 29th cycle. After this point, training stops because there is no further improvement in the validation loss value.

This trajectory indicates that the encoder–decoder pair quickly learns coarse cloud structure representations (e.g., large cloud clusters and open sky regions) during early training, then refines fine details (e.g., cloud edge textures and thin cirrus patterns) as training progresses. The final MSE of 0.0037 corresponds to a root mean square error of approximately 0.06 in the normalized [0,1] mask space, representing an average deviation of approximately 6% per pixel between the input and reconstructed masks.

Figure 5b shows that the commitment loss $L_{\mathrm{VQ}}$ decreases from 0.0021 to 0.0017 and stabilizes after the 7th epoch. This model reflects the convergence of the code book learning process via exponential moving average: early training involves frequent reassignment of encoder outputs to different codebook inputs as the model explores the latent space, while later training exhibits stable token assignments after the code book captures the diverse vocabulary of recurrent cloud models. The low final commitment loss (0.0017) indicates minimal inconsistency between continuous encoder outputs and their closest discrete code book neighbors, confirming that quantization does not introduce significant information bottlenecks.

The synchronized convergence of both loss components after epoch 7 suggests that the VQ-VAE reaches a stable equilibrium where reconstruction quality and codebook consistency are jointly optimized. This stability is critical for downstream transformer training (Stage 3), as inconsistent token assignments would cause distribution shift and prevent the autoregressive model from learning reliable temporal patterns.

### 5.5. Model Performance

To measure the performance of the proposed pipeline, its ability to predict the next time step (t+1) is evaluated on the test set when four context frames (t−3,t−2,t−1,t) are provided. The choice of T=4 context frames follows the established convention in the video prediction literature [57], where a four-frame window has been shown to provide sufficient short-range temporal context for next-frame prediction while remaining computationally tractable. This single-step accuracy serves as a baseline for assessing subsequent multi-step rollout degradation.

The model was evaluated on 122 sequences, each containing an average of 680 consecutive frames. The model’s performance was compared with deep learning and traditional baseline models. Optical flow estimates per-pixel displacement between consecutive frames using the Lucas–Kanade method [61] and warps the most recent frame along the estimated flow field to produce a prediction. Phase correlation computes the cross-power spectrum of two successive frames in the Fourier domain to recover a global translation vector, which is then applied to the last context frame. Both methods are parameter-free and serve as classical baselines.

ConvLSTM [14] embeds convolutional operators within long short term memory (LSTM) gate functions, enabling spatiotemporal feature learning with locally consistent dynamics. It is trained with the same BCEWithLogitsLoss objective and Adam optimiser ($\eta =10^{-3}$) as the proposed model, using a 2-layer architecture with 64 hidden channels. Predictive Recurrent Neural Network (PredRNN) [62] extends ConvLSTM via a zigzag spatiotemporal memory that propagates information across both time steps and network depth. U-Net Predictor [63] is an encoder–decoder network with skip connections that receives all four context frames concatenated along the channel dimension as input and directly regresses the next cloud mask. The models used for comparison are trained using the same training settings as the proposed architecture.

#### Single-Step Prediction Performance

The proposed pipeline delivers consistent improvements across all metrics reported in Table 1, compared to both classical motion estimation methods and deep learning models. The performance improvements observed in the proposed pipeline are based on the complementary roles of the proposed architectural components and have been examined in detail through an ablation study.

**Figure 6:**
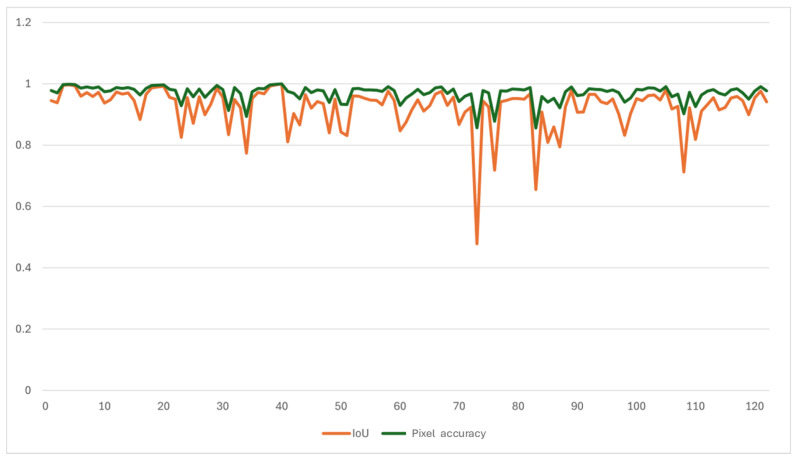
Single-step prediction performance of 122 different sequences.

**Figure 7:**
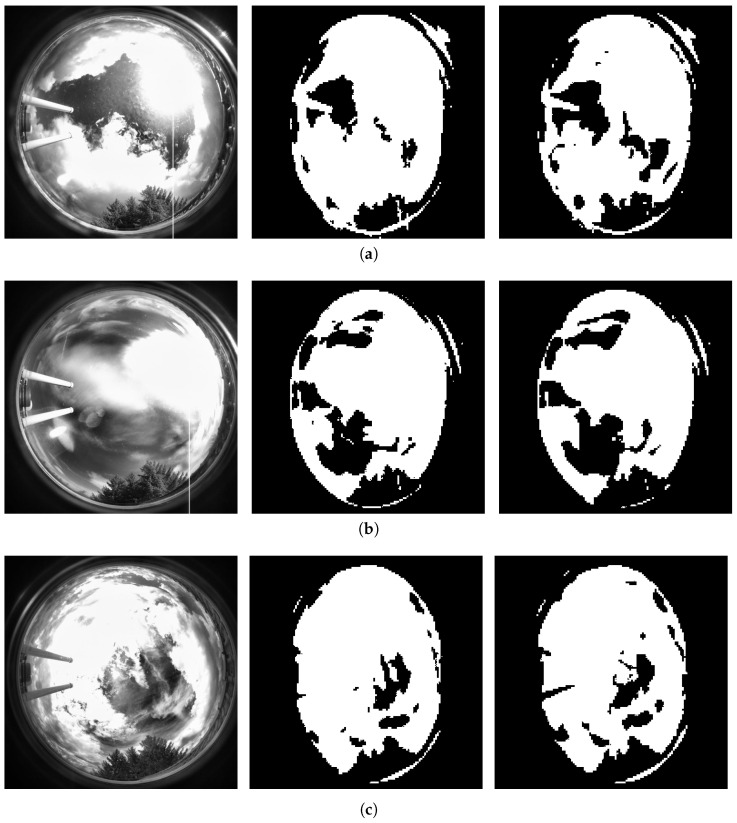
Single-step prediction samples. (**a**) 11 AM: actual image (**left**), ground-truth mask (**middle**), and single-step prediction (**right**) with IoU = 0.8996 and pixel accuracy = 0.9561. (**b**) 10 AM: actual image (**left**), ground-truth mask (**middle**), and single-step prediction (**right**) with IoU = 0.924842 and pixel accuracy = 0.970657. (**c**) 04 PM: actual image (**left**), ground-truth mask (**middle**), and single-step prediction (**right**) with IoU = 0.907641 and pixel accuracy = 0.961639.

Figure 6 provides a frame-by-frame breakdown of IoU and pixel accuracy across the 122 test sequences, revealing temporal variability in prediction quality. Frames with lower IoU (typically <0.85) correspond to rapid cloud formation events, sunset/sunrise transitions with complex lighting, while high-quality predictions (IoU > 0.95) occur during stable cloud configurations with persistent motion patterns. Figure 7 presents qualitative examples from three representative time periods, showing (left to right): original grayscale sky images, ground-truth cloud masks, and model predictions. Visual inspection confirms that the model accurately captures cloud spatial distribution, preserves boundary sharpness, and maintains structural coherence, with minor discrepancies primarily occurring at the regions with sunshine.

### 5.6. Ablation Studies

#### 5.6.1. Role of the Segmentation Module

The 9.7% drop in IoU and 8 point decrease in SSIM observed in the *Lightweight CNN* variant demonstrates that prediction quality is fundamentally limited by the accuracy of the segmentation in the previous stage. The residual encoder of UCloudNet, that inspired this work, applies multi-resolution feature fusion and enables the network to learn semantically meaningful features at 1/2 and 1/4 output scales via auxiliary losses [37]. When this stage is skipped, blurred mask boundaries propagate as corrupted token assignments into the VQ-VAE codebook, and this cascading error manifests in every downstream metric. This behavior is consistent with the principle that perceptual noise upstream of the discrete tokenization stage increases the codebook entropy and degrades the quality of the token sequences fed to the predictor [20].

#### 5.6.2. Role of the Transformer Predictor

Replacing the Transformer with a simpler predictor results in a 5.5 point drop in IoU and a 5.7 point decrease in SSIM; this demonstrates that local spatial operators alone are insufficient for capturing the long-range temporal dependencies specific to cloud advection across four input frames. Unlike recurrent models, which are limited to sequential computation via gradient paths, the Transformer’s full self-attention mechanism attends jointly over all temporal tokens collectively, enabling the model to represent non-local atmospheric circulation patterns that extend beyond the effective receptive field of convolutional or recurrent kernels [21]. The discrete latent vocabulary produced by VQ-VAE fits well with the Transformer sequence modeling framework by converting continuous cloud masks into a finite codebook. The prediction task becomes an autoregressive token classification problem where Transformers show strong generalization in spatiotemporal prediction tasks [20,21].

#### 5.6.3. Codebook Ablation

In the codebook ablation study, codebooks of varying sizes with 64 embedding dimensions and codebooks with 1024 embeddings of varying embedding dimensions were evaluated. For this study, models were retrained on 20% of the training data, and performance was evaluated using five consecutive frames per sequence. As shown in Table 2, which presents the results across different codebook configurations, the highest utilization rate was achieved by the codebook consisting of 128 embeddings, each with 64 embedding dimensions. No codebook collapse was observed for this configuration. The proposed codebook structure—consisting of 1024 embeddings, each with 64 embedding dimensions—ranked second in utilization rate and utilized only 30% of the total tokens; however, it yielded slightly better reconstruction performance (0.3% higher Reconstruction IoU) compared to the *cb128* configuration, where *cb128* denotes a codebook with *128* embeddings, each of 64 dimensions. While the smaller codebook proved to be more efficient in terms of compactness, the configuration achieving the highest overall performance was selected for this study.

#### 5.6.4. Transformer Model Ablation

The most commonly encountered architecture in the literature is the 8-layer transformer, consistent with the original architecture [21]. A comparative study was conducted to evaluate the performance of 8-layer and 6-layer transformer models. As shown in Table 3, the 6-layer architecture achieved marginally better performance. Furthermore, the 6-layer transformer was adopted in this study to reduce overall computational complexity.

### 5.7. Comparison with Baselines

#### 5.7.1. Classical Motion Estimation

Optical flow and phase correlation are the most commonly used signal processing methods for estimating cloud motion. Optical flow is based on the assumption of luminance constancy meaning that pixel intensity remains constant between consecutive frames [64]. However, since cloud regions exhibit changes in radiative intensity due to variations in solar angle and atmospheric scattering while simultaneously undergoing translational motion and morphological deformation, this assumption loses its validity in satellite and ground-based sky images [65,66].

#### 5.7.2. Deep Learning Models

ConvLSTM captures spatiotemporal dependencies by embedding convolutional operators within LSTM gate functions, which makes it highly suitable for locally consistent dynamics [14]. PredRNN extends this paradigm via a zigzag spatiotemporal LSTM that spreads memory across both time steps and network depth, partially mitigating the accumulated error of deep recurrent stacks [62]. The advantage of PredRNN over ConvLSTM ΔIoU=0.003 is consistent with this architectural improvement but both models remain constrained by convolutional receptive fields. Temporal dynamics are modeled through local spatial operations, and large-scale advection models should propagate gradually through recurrent hidden states rather than being handled globally.

The superiority of the proposed model over PredRNN, particularly the reduction in MAE from 0.017 to 0.011 and the 0.006 increase in SSIM, can be attributed to the Transformer’s ability to establish direct attention paths between any two spatial tokens across its entire temporal context window; a capability that recurrent architectures can only approximate imperfectly through state propagation [21]. The relatively weaker performance of U-Net Predictor [63] supports the idea that, without a dedicated temporal memory or attention mechanism, encoder–decoder architectures lose the sequential order and dynamic structure of temporal context by combining four input frames into a single hidden representation.

### 5.8. Multi-Step Forecasting and Error Propagation

**Figure 8:**
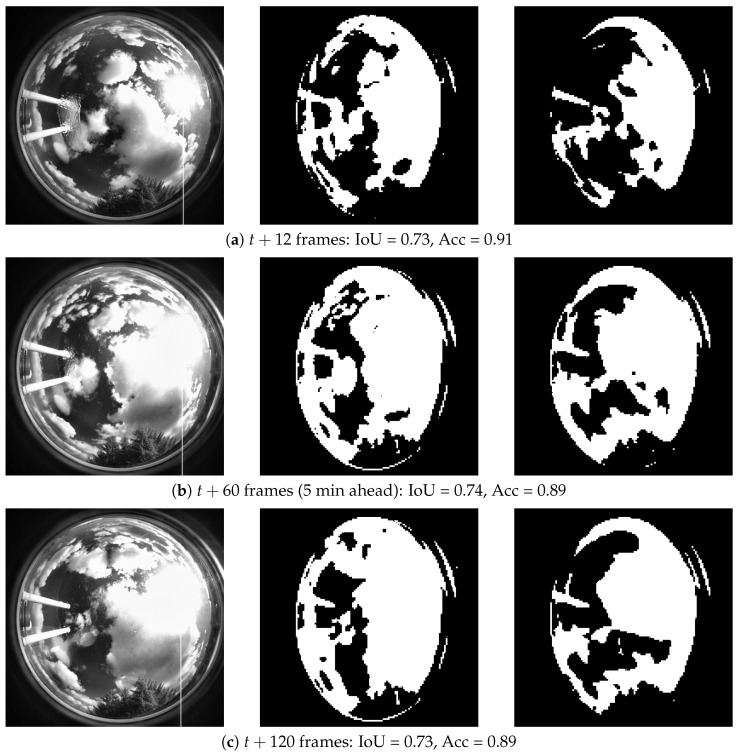
Qualitative examples of autoregressive cloud mask predictions at three forecast horizons. Each row shows (**left** to **right**): original sky image, ground-truth mask, and predicted mask.

To ensure temporal stability, long forecasting using recursive rollout is evaluated. Long-term forecasts were generated by autoregressively predicting up to 120 future frames (10 min), where each newly predicted frame is appended to the input sequence for the subsequent prediction step. Due to error accumulation specific to autoregressive feedback loops, accuracy gradually decreases over the prediction horizon; however, the model provides meaningful spatial consistency as confirmed by IoU values above 0.65 for up to 10 min. Multi-step rollout performance across different horizons is summarized in Table 4. As seen in the table, as the prediction time increases, a certain decline in model performance begins. The reason for this is that the model attempts to continue its prediction without seeing any original images during the 10 min prediction period. In other words, to predict the 120th image, the model generates 119 frames and achieves a 0.63 IoU and 0.86 pixel accuracy value at the end of the period. At last, Figure 8 compares the actual image, ground-truth cloud masks and the corresponding predictions for the next 1, 5, and 10 min.

### 5.9. Model Complexity Comparison

Table 5 summarizes the computational complexity of all evaluated models in terms of trainable parameters, inference latency, peak GPU memory consumption, and Giga Floating Point Operations Per Second (GFLOPS), which quantifies how many billions of arithmetic operations, specifically additions and multiplications, are needed to complete one forward pass through a neural network.

The proposed pipeline contains 29.4 M trainable parameters and requires 180.6 GFLOPS per inference step, consuming approximately 3.7 GB of GPU memory, with a measured latency of 91.5 ms on a single Nvidia A100 GPU. In particular, despite having significantly fewer parameters (1.5 million and 2.3 million, respectively), ConvLSTM and PredRNN exhibit significantly higher computational costs of 602.8 and 1200.7 GFLOPs, respectively. This is due to the recurrent spatial convolution operations; these operations process full H×W feature maps repeatedly across multiple time steps, leading to high FLOP values despite the compact number of parameters.

The U-Net Predictor achieves the lowest latency (2.1 ms) and GFLOPs (27.5) among learned models, but its inferior forecasting performance indicates that raw spatial regression without discrete latent modeling is insufficient for accurate cloud motion forecasting.

Considering performance efficiency, the proposed model delivers the best performance with an *IoU/GFLOPs* ratio of $5\times 10^{-3}$, despite consuming several times more GPU memory than competing models.

### 5.10. Uncertainty Analysis

Modeling uncertainty in cloud evolution is a key feature of the proposed framework, enabling the model to express a distribution of possible outcomes rather than a single deterministic prediction. Following [67], we distinguish three sources of predictive uncertainty in cloud motion forecasting: *epistemic uncertainty* arising from model parameters, *aleatoric uncertainty* arising from input data noise, and *chaotic uncertainty* intrinsic to atmospheric dynamics [68].

#### 5.10.1. Uncertainty Source Decomposition

The following estimation strategies are used to identify sources of uncertainty:

- Epistemic uncertainty is estimated via Monte Carlo (MC) Dropout [69]. Dropout layers are kept active at inference time, and K=10 stochastic forward passes are executed with deterministic (argmax) token decoding. The pixel-wise variance across these passes reflects uncertainty attributable to model parameters.
- Aleatoric uncertainty is estimated by input perturbation. Independent Gaussian noise (σ=0.05) is added to each context frame across *K* trials, with deterministic decoding applied in each trial. The resulting pixel-wise variance reflects the model’s sensitivity to input observation noise.
- Chaotic uncertainty is approximated as the residual component:
  (10) $$U_{\mathrm{chaotic}}\left(i,j\right)=\max\;\left(U_{\mathrm{total}}\left(i,j\right)-U_{\mathrm{epistemic}}\left(i,j\right)-U_{\mathrm{aleatoric}}\left(i,j\right),\;0\right),$$
  where $U_{\mathrm{total}}$ is estimated via temperature sampling. This residual captures the irreducible stochasticity of cloud motion that cannot be attributed to model parameters or input noise.

**Figure 9:**
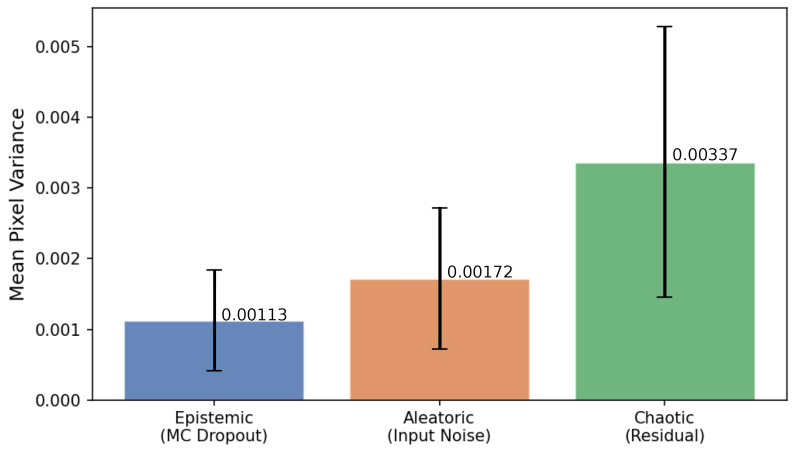
Decomposition of predictive uncertainty into epistemic, aleatoric, and chaotic components, measured as mean pixel variance.

Figure 9 shows the average pixel variance contributed by each source in the test set. Chaotic uncertainty accounts for the largest share (54%), followed by the aleatoric (28%) and epistemic (17%) components. The dominance of chaotic uncertainty is consistent with the inherently unpredictable nature of atmospheric dynamics on short timescales [68], and the low epistemic contribution indicates that the model was well-trained with limited parameter uncertainty.

#### 5.10.2. Uncertainty–Error Correlation Analysis

In this study, uncertainty is estimated by generating 10 future predictions for the same input sequence across all input sequences and measuring how much these predictions differ from one another at each pixel. When these outcomes vary strongly at certain locations, the model reports higher uncertainty there, indicating less confidence in the prediction.

Let ${\hat{Y}}^{\left(k\right)}=\left[{\hat{Y}}_{i,j}^{\left(k\right)}\right]\in {\left[0,1\right]}^{H\times W}$ denote the *k*-th probabilistic cloud mask prediction, where k=1,…,K and (i,j)∈{1,…,H}×{1,…,W} denotes pixel coordinates. Let $Y=\left[Y_{i,j}\right]\in {\left\{0,1\right\}}^{H\times W}$ denote the corresponding binary ground-truth cloud mask.

Pixel-wise predictive uncertainty is computed as the variance across multiple sampled predictions [69]:

(11) $$U\left(i,j\right)=\mathrm{Var}\left({\hat{Y}}_{i,j}^{\left(1\right)},{\hat{Y}}_{i,j}^{\left(2\right)},\ldots ,{\hat{Y}}_{i,j}^{\left(K\right)}\right),$$

where (i,j) denotes the pixel location. Then the prediction error is quantified using the IoU metric between the predicted mask and the ground truth. For correlation analysis, the error is defined as

(12) $$E=1-\mathrm{IoU}(\hat{Y},Y).$$

The quality of the uncertainty estimate was evaluated at two different levels of detail. At the pixel level, the estimated uncertainty exhibits a Pearson correlation of r=0.299 and a Spearman rank correlation of ρ=0.365 with the pixel-based binary prediction error; this indicates a moderate yet consistent relationship between local uncertainty and local accuracy. At the sequence level, the sum of the average uncertainty and IoU error per sequence yields a significantly stronger Spearman rank correlation of ρ=0.873 (p≈0.031); this confirms that sequences with higher prediction uncertainty reliably align with those having lower prediction quality. The near-zero Pearson coefficient at this level (r=0.031, p=0.736) indicates that the relationship is monotonic but not linear; this is consistent with the limited nature of the IoU metric.

**Figure 10:**
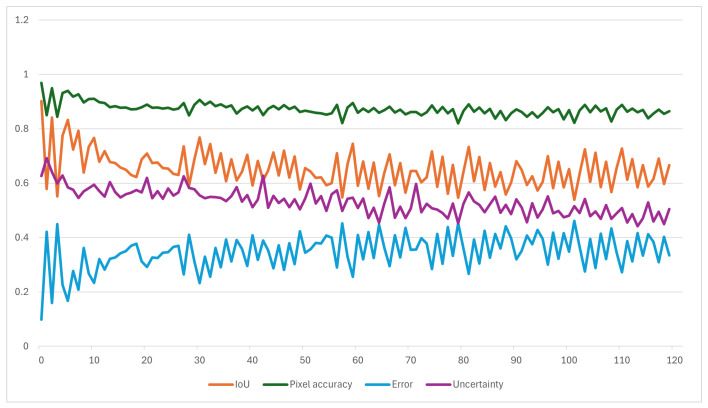
Intersection over union, pixel accuracy, uncertainty, and error over a 120-step autoregressive cloud mask forecasting horizon.

Rather than producing overly confident predictions with high probability values but low accuracy, the proposed model exhibits a lower correlation between the predicted cloud probability and the actual cloud masks, indicating that prediction reliability appropriately decreases in uncertain regions. Instead of generating spurious high-frequency details under uncertain conditions, the model produces outputs with broader spatial distributions that are consistent with increased ambiguity in cloud evolution as reflected in the variance across sampled predictions. This property is important for practical applications, as it enables the model both to predict cloud movement and to signal when its forecasts may be less reliable. Figure 10 illustrates the model’s performance over a 120-step (10 min) forecast horizon on a sequence. As accuracy declines due to error accumulation, uncertainty estimates decrease correspondingly after step 50, indicating that the model correctly signals reduced confidence at longer forecast horizons.

## 6. Conclusions

This study presents a modular deep learning framework for short-term cloud motion using ground-based all-sky images. The system is based on a three-stage process: (1) A lightweight Convolutional Neural Network segments cloud regions and generates probabilistic masks, (2) a Vector Quantized Variational Autoencoder compresses these masks into discrete token sequences, and (3) a GPT-style autoregressive transformer predicts future token sequences based on temporal context. The framework is trained on 103,304 images from the CASCAR dataset [55] selected from a custom dataset and evaluated on held-out test sequences in both single-step and multi-step autoregressive prediction scenarios.

Quantitative evaluation shows that the model achieves an average intersection-over-union ratio of 0.92 and pixel accuracy of 0.96 for one-step (5 s forward) predictions on the test set. Under an autoregressive rollout extending up to 120 steps (10 min), performance degrades due to error accumulation, with IoU dropping to 0.65 at the final prediction horizon and pixel accuracy falling to 0.80. Specifically, while 80% of single-step predictions maintain IoU above 0.90, 89% of squares in the entire 120-step rollout maintain pixel accuracy above 0.85. Uncertainty measurement via token-level prediction-based entropy shows a positive correlation with prediction error (1 - IoU) and confirms that the model reliably signals reduced reliability at longer prediction horizons. These results demonstrate that the framework is suitable for operational nowcasting applications requiring reliable predictions for up to 10 min.

The fundamental limitation of the current approach is that the quality of predictions longer than 10 min decreases due to the accumulation of errors in the autoregressive feedback loop. While discrete token representation is computationally efficient, it produces small correction artifacts at cloud boundaries due to 8× spatial downsampling.

Additionally, the model operates in an entirely data-driven way without explicitly incorporating atmospheric physics (e.g., wind speed fields and mass conservation constraints), which may limit extrapolation to cloud motion models poorly represented in the training distribution.

To evaluate the effectiveness of the proposed method, various ablation studies were conducted using traditional and deep learning-based prediction models commonly used in the literature, and these were tested comparatively on the same dataset [14,62,63]. The quantitative results obtained demonstrate that the proposed pipeline exhibits significantly better prediction performance compared to all comparison models.

In future research, integrating existing deep learning models trained on all-sky camera images with satellite data and wind measurements within a multimodal framework has the potential to significantly improve prediction accuracy, and model robustness and may enable the expansion of the forecasting horizon. Additionally, transformations applied to the inputs of deep learning models based on the geographic coordinate system can enable the model to achieve broader applicability without incurring additional computational overhead.

## Figures and Tables

**Table 1:** Quantitative comparison of the proposed model against ablated variants and state-of-the-art baselines.

Model	IoU	F1-Score	Pix. Acc.	MAE	MSE	SSIM
Full Model	**0.920 **	**0.935**	**0.948**	**0.011**	**0.011**	**0.911**
w/o CNN	0.837	0.888	0.928	0.022	0.002	0.835
w/o Transformer	0.865	0.904	0.925	0.035	0.035	0.854
Optical Flow	0.880	0.913	0.932	0.028	0.025	0.867
Phase Correlation	0.861	0.902	0.923	0.036	0.036	0.852
ConvLSTM [14]	0.908	0.930	0.943	0.017	0.015	0.904
PredRNN [62]	0.911	0.930	0.943	0.017	0.015	0.905
U-Net Predictor [63]	0.903	0.925	0.941	0.019	0.017	0.897

**Table 2:** Ablation study of the different size of codebook. In variant names, “cb” represents codebooks of varying lengths with an embedding dimension of 64, while “emb” represents codebooks with an number of embedding of 1024.

Variant	Number of Embeddings	Embedding Dimension	Codebook Util. Rate (%)	Number of Active Codebook Entries	Recon. IoU	Recon. MAE	Pred. IoU	Pred. F1-Score
cb128	128	64	**100**	128	0.968	0.002	0.936	0.952
cb256	256	64	9.38	24	0.961	0.005	0.930	0.942
cb512	512	64	5.08	26	0.963	0.004	0.932	0.950
Proposed	1024	64	30.86	316	**0.971**	**0.001**	**0.938**	**0.953**
Architecture								
emb32	1024	32	4.39	45	0.963	0.004	0.934	0.951
emb128	1024	128	2.15	22	0.955	0.008	0.925	0.946

**Table 3:** Performance comparison of 6 and 8-layer transformer models.

Layer Count	IoU	F1-Score	Pix. Acc.	MAE	MSE	SSIM
6-layer	**0.920**	**0.935**	**0.948**	**0.011**	**0.011**	**0.911**
8-layer	0.919	0.935	0.947	0.011	0.011	0.910

**Table 4:** Mean IoU and accurcay performance values every three minutes during the model’s 10 min prediction process.

Horizon Slot	Mean IoU	Mean Accuracy
(0, 3) min	0.6920	0.8915
(3, 6) min	0.6410	0.8680
(3, 10) min	0.6327	0.8615

**Table 5:** Model complexity comparison table.

Model	Params (M)	GFLOPs	Latency (ms)	GPU Mem (MB)
Proposed Pipeline	29.43	180.6	91.5	3699
ConvLSTM [14]	1.50	602.8	10.7	622
PredRNN [62]	2.31	1200.7	27.0	756
U-Net Predictor [63]	7.76	27.5	2.1	186

## Data Availability

The dataset used in this study is openly available in Zenodo. https://doi.org/10.5281/zenodo.18657514 accessed on (16 February 2026).

## Abbreviations

The following abbreviations are used in this manuscript:

PV	Photovoltaic
CNN	Convolutional Neural Network
ConvLSTM	Convolutional Long Short-Term Memory
VQ-VAE	Vector-Quantized Variational Autoencoder
RGB	Red-Green-Blue
IoU	Intersection over Union
PhyCell	Physics-informed Cell
SWINySEG	Singapore Whole sky Nychthemeron Image Segmentation
U-Net	U-Net
RNN	Recurrent neural network
PDE	Partial differential equations
INDI	Instrument Neutral Distributed Interface
GPT	Generative Pre-trained Transformer
CASCAR	Clausthal All Sky Camera Recordings
BCEWithLogitsLoss	Binary Cross-Entropy with Logits Loss
MAE	Mean Absolute Error
MSE	Mean Squared Error
SSIM	Structural Similarity Index Measure
PredRNN	Predictive Recurrent Neural Network
LSTM	Long short-term memory
GFLOPs	Giga Floating Point Operations Per Second
MC	Monte Carlo
